# Recent advances in amyotrophic lateral sclerosis

**DOI:** 10.1007/s00415-016-8091-6

**Published:** 2016-03-30

**Authors:** Nilo Riva, Federica Agosta, Christian Lunetta, Massimo Filippi, Angelo Quattrini

**Affiliations:** Neuropathology Unit, INSPE and Division of Neuroscience, Department of Neurology, Institute of Experimental Neurology, San Raffaele Scientific Institute, Via Olgettina 48, 20132 Milan, Italy; Neuroimaging Research Unit, Division of Neuroscience, Department of Neurology, Institute of Experimental Neurology, San Raffaele Scientific Institute, Milan, Italy; NEuroMuscular Omnicentre (NEMO), Niguarda Ca Granda Hospital, Milan, Italy

**Keywords:** Motor neuron disease, Pathogenesis, ALS genetics, Clinical management, Neuroimaging

## Abstract

ALS is a relentlessly progressive and fatal disease, with no curative therapies available to date. Symptomatic and palliative care, provided in a multidisciplinary context, still remains the cornerstone of ALS management. However, our understanding of the molecular mechanisms underlying the disease has advanced greatly over the past years, giving new hope for the development of novel diagnostic and therapeutic approaches. Here, we have reviewed the most recent studies that have contributed to improving both clinical management and our understanding of ALS pathogenesis.

## Introduction

Motor neuron disease (MND) embraces a heterogeneous group of neurological disorders defined and characterized by the degeneration of motor neurons. With an incidence of ~2.16 per 100,000 person-years and a median survival time of 3–5 years, amyotrophic lateral sclerosis (ALS) is the most common and severe form, involving both lower (LMN) and upper motor neurons (UMN). This review summarizes the recent highlights in ALS research published in the Journal of Neurology and other relevant scientific Journals during the last 18 months. For an overview of MND and ALS, readers are referred to other more general reviews [1–3].

## Proposed disease mechanisms

Significant advances have been made in our understanding of MND pathogenesis, believed to be a complex disease involving an intricate combination of exogenous environmental factors and common and rare genetic variations [4]. About 10 % of ALS cases are classified as familial (FALS), whereas the remaining 90 % appear to be sporadic (SALS) and randomly occurring in the community. While a genetic etiology may now be identified in two-thirds of FALS and ~10 % of apparently sporadic cases, the role of genetic and environmental factors in sporadic cases is still unknown [5–7].

### Molecular genetics

Since the identification in 1993 of mutations of the SOD1 gene as being responsible for some forms of autosomal dominant FALS [8], more than 30 other genes linked to ALS have been identified [9].

The identification of TDP-43 protein as a major component of the neuronal inclusions of both ALS and fronto-temporal dementia (FTD) not only provided pathological evidence that these apparently distinct conditions constitute a disease spectrum but also led to the identification of a mutation of TARDBP gene as responsible for ~4 % of FALS cases. Subsequent studies demonstrated that mutations in the TARDBP gene may be responsible not only for FALS and a small percentage of SALS cases, but also for FTD, FTD-ALS and ALS-FTD cases [10, 11]. Interestingly, a recent study and review of the literature, demonstrated a predominant temporal lobe involvement and semantic dementia phenotype in a high percentage of FTD patients with this mutation [12]. Mutations in TDP-43 and in the fused in sarcoma genes (FUS) [13], both RNA-binding proteins and sharing functional homologies, have highlighted the importance of RNA processing in ALS pathogenesis [14, 15]. Recently, mutations in Matrin 3 (MATR3), an RNA- and DNA-binding protein that interacts with TDP-43 and earlier described as a cause of distal myopathy [16], have been identified as a cause of FALS [17], providing further evidence of the role of aberrant RNA processing in motor neuron degeneration.

Discovered in 2011 [18, 19], the C9orf72 repeat expansion represents not only the most common genetic cause of FALS of European descent (more than one-third), but also accounts for a significant percentage of apparently sporadic ALS cases (~7 %). Moreover, C9orf72 repeat expansions account for many familial FTD cases (~25 %) and genetically explains the overlap between these two clinical syndromes [6, 20]. Although the most frequent phenotypes are ALS, the behavioral variant FTD (bvFTD) or ALS/FTD the presentations associated with C9orf72 repeat expansion may be extremely heterogeneous, in regard to disease progression rate, neuropsychiatric, behavioural and motor features [21]. The potential role of C9orf72 repeat expansions in other neurodegenerative disorders, such as Parkinson Disease (PD) or atypical parkinsonism is still to be elucidated [22]. A recent study revealed intermediate 20–30 repeat expansions in 4.3 % of patients presenting with non-classical atypical parkinsonism with FTD-like dementia or without dementia and with an upper MND-like phenotype [23], suggesting a potential pathogenetic role for intermediate repeat sizes, in line with some earlier reports [24–26]. C9orf72 repeat expansions have also been suggested by recent studies as a possible genetic cause of Huntington disease phenocopy syndrome, further expanding its potential spectrum of presentation [27–29].

Although the mechanism leading to neurodegeneration in C9orf72 expansions is not fully understood [21], potential mechanisms include loss of C9orf72 protein and function, RNA toxicity and sequestration of RNA-binding proteins [30], or alternatively toxicity from the dipeptide repeat (DPR) proteins, which are the pathologic hallmark of C9orf72 expansion-related neuronal inclusions [31].

Importantly, the identification of C9orf72-related ALS has encouraged scientists to search for other repeat-expansions. While ATXN-2 (SCA2) and ATXN-1 (SCA1) PolyQ intermediate expansions have been previously reported to be independently associated with an increased risk for ALS [32–34], co-occurrence of ALS and SCA1 within a family carrying an intermediate ATXN-1 poly-Q expansion has been recently reported, reinforcing the putative pathogenic link between the two disorders [35].

Further evidence of association between MND and FTD has been more recently provided by the demonstration that loss of function (LoF) mutations of the TBK1 gene, encoding the TANK-binding kinase 1, causes a dominant form of ALS and FTD which could potentially account for up to 4 % of FALS cases [36, 37]. Mutations would result in an impaired TBK1 interaction with optineurin and sequestosome-1 (also known as ubiquitin-binding protein p62), both implicated in ALS pathogenesis [38, 39]. All these genes are involved in one common pathway of autophagy regulation [36, 37].

An even broader range of clinical presentations has been recently related to mutations in the coiled-coil-helix-coiled-coil-helix domain containing 10 (CHCHD10) gene, encompassing not only ALS without cognitive impairment, ALS/FTD or FTD (~1 % of cases), but also parkinsonism, cerebellar ataxia and mitochondrial myopathy [40–43]. These findings support the hypothesis that mitochondrial dysfunction may be implicated in the pathogenesis of ALS and several other neurodegenerative disorders [44].

Recently, identification of putative mutations in the profilin 1 (PFN1) [45] and TUBA4A [46] genes has indicated that defects in neuronal cytoskeleton architecture may also contribute to MND pathogenesis.

PFN1 mutations were initially identified using an exome-sequencing approach in 2.6 % of FALS cases [45]. A mutational analysis of the PFN1 gene was carried out in a Catalan cohort of 42 FALS and 423 SALS patients [47]. No PFN1 mutations were identified. This is consistent with other negative studies in other populations [48–51], suggesting that PFN1 is not a major ALS-causing gene.

An excess of patient variants (7/635) in TUBA4A gene was first identified by an exome-wide burden analysis study of rare variants in FALS index cases [46]. A second study identified four novel TUBA4A variants with predicted deleterious effects in a cohort of 1106 SALS of Italian origin, supporting the role of TUBA4A gene in ALS [52]. However, further confirmative and functional studies are needed before any individual TUBA4A variant can be implicated in ALS [53].

During the last few years, several genome-wide association studies (GWAS) have been used not only in order to identify new risk loci but also to discover genetic variants that may influence ALS phenotype, for example age at onset or prognosis. Although most of these associations still need replication [7], a recent study confirmed that the UNC13A rs12608932 is not only a risk factor for ALS in the Spanish population, but would also appear to be a modifying factor for survival and disease progression rate [54]. This is consistent with previous reports [55–58].

## Environmental factors

Various environmental factors have been proposed to be associated with ALS, such as smoking, antioxidant intake, body mass index, physical exercise, head trauma, metabolic and inflammatory states, cancer, and occupational or environmental exposures to electromagnetic fields, metals or pesticides [59]. However, the only established risk factors to date are older age, male gender, and a family history of ALS [60].

Potential associations between medical conditions and ALS was explored by a recent population-based case–control study performed in the Netherlands, including 722 sporadic ALS patients [61].

A significant association between head trauma and ALS susceptibility (OR 1.95, 95 % CI 1.11–3.43, *p* = 0.02), was found, in line with previous reports [62–65]. In contrast, hypercholesterolemia (OR 0.76, 95 % CI 0.63–0.92, *p* = 0.006) and the use of statins (OR 0.45, 95 % CI 0.35–0.59, *p* = 1.86 × 10^−9^) were instead associated with a decreased risk of ALS, in line with previous reports but in contrast with other studies, that reported either higher [66] or unaltered [67] lipid levels in ALS patients compared to controls. Moreover, the use of immunosuppressive drugs were associated with a decreased risk of ALS (OR 0.26, 95 % CI 0.08–0.86, *p* = 0.03), in contrast with two previous hospital-based reports [68, 69].

One of the key clinical and pathological observations is that ALS onset is focal and discrete, subsequently followed by progressive spread of the neurodegenerative process [70].

While the split hand syndrome is a recognized distinctive clinical and neurophysiological phenomenon in ALS [71], defined by dissociated wasting of the hypothenar and thenar muscles, a recent study has also demonstrated dissociated involvement of lower limb ankle muscles. These observations suggest underlying selective differences in susceptibility to degeneration or disequilibrium of excitatory inputs within motor neuronal subsets [72].

A potential link between exogenous factors and focality at onset has been suggested by a recent retrospective observational study, identifying18 out of 1835 subjects who developed MND after frontal contusions or other frontal intracranial lesions; interestingly, symptom onset started in a body region reflecting the damaged cortical area in the vast majority (15/18) of the patients [73]. Moreover, ALS has recently been linked to previous embolization of a subgroup of cerebral arteriovenous malformations (AVM) with perinidal angiogenesis, suggesting a link between altered angiogenesis and low VEGF production and ALS development [74].

## ALS phenotype heterogeneity

The ALS clinical spectrum includes extremely heterogeneous and complex phenotypes marked by a varying involvement of upper and lower motor neurons, site of onset and rate of progression [70]. Recognized MND phenotypes include classic, bulbar, flail arm, flail leg, pyramidal and respiratory ALS, primary lateral sclerosis (PLS), characterized predominantly by pure/predominant UMN degeneration and progressive muscular atrophy (PMA), characterized by pure/predominant LMN degeneration [75]. The motor phenotype is so heterogeneous that it may overlap with other diseases of the MND spectrum, such as hereditary spastic paraplegia or distal motor neuropathy (dSMA). Noteworthy, recent studies have demonstrated that causative genes identified so far are not uniquely associated with a single clinical form but may be responsible for different phenotypes within the MND spectrum, ranging from predominant lower to upper motor neuron involvement. For instance, recent reports have highlighted how such genes as DCTN1 (dynactin 1) [76–78], GDAP1 (ganglioside-induced differentiation associated protein 1) [76, 79, 80], DYNC1H1 (Dynein, cytoplasmic 1, heavy chain 1) [81, 82], KIF5A (Kinesin Heavy Chain Isoform 5A) [81, 83], NEFH (neurofilament heavy chain gene) [84, 85] have all been associated with a wide range of phenotypes, ranging from ALS to hereditary spastic paraparesis (HSP) [86], dSMA or even classic Charcot–Marie–Tooth disease [76, 81, 82].

Besides “pure” motor symptoms, a varied degree of extra-motor involvement may be observed in MND patients. Following early clinical descriptions, the identification of TDP43-positive ubiquitinated cytoplasmic inclusions in almost all ALS cases and more than half of FTD patients [87], has rekindled interest in the overlap between these neurodegenerative syndromes [1]. This is further supported by neuroradiological studies [88] and recent advances in ALS and FTD genetics, as described above. While cognitive abnormalities characterized by executive and behavioral abnormalities, language and memory impairment, may be detected in up to 50 % of ALS patients [89, 90], at the extreme end of the spectrum, overt FTD may be diagnosed in up to 15 % of ALS patients [91, 92]. In line with this, a recent study performed in 126 Chinese SALS patients found that 46.0 % of subjects showed varyingi degrees of frontal behavioral changes based on the frontal behavioral inventory (FBI) score, while the prevalence of frontal lobe impairment, assessed with the frontal assessment battery (FAB), was as high as 32.5 %, with “lexical fluency deficit” being the highest affected domain (30.7 %) [93]. Impaired language, and more specifically significant difficulty with action verb associativity judgments in ALS has also been confirmed by a more recent study [94]. Executive dysfunction may also predict social cognition impairment, which refers to the capability of encoding and decoding socially salient information, such as the emotions and intentions of others [95]. Since these processes are considered to be supported by a frontostriatal network, these data further support a cognitive continuum between ALS and FTD [96, 97]. Importantly, it has also been recently shown that cognitive and behavioural status is a major predictor of caregiver burden [98].

Besides cognitive impairment, concomitant sensory nervous system involvement has also been previously independently reported in ALS [99]. Interestingly, a recent report has suggested that patients with spinal-onset but not those with bulbar-onset ALS have a concomitant distal small-fibre neuropathy [100]. This finding is in keeping with previous reports [101] and may also be viewed in light of the recently proposed focal-spreading model of neurodegeneration in ALS [102, 103].

## Prognosis

ALS phenotypic heterogeneity is reflected in the variability of ALS prognosis: although approx. one half of patients die within 30 months of symptom onset, ~20 % of patients survive between 5 and 10 years [104]. The identification of the key prognostic factors is therefore important for appropriate timing of medical interventions and stratification in clinical trials.

Notably, a simple prognostic algorithm was recently proposed. Employing a multivariate model, the negative prognostic indicators included in the model were: bulbar and respiratory site of disease onset, executive dysfunction, and ALSFRS-R slope prior to first evaluation [105]. These results are in keeping with previous reports [104, 106–110], but unlike in these previous studies, older age of onset and female gender were not confirmed as predictors of poor prognosis [111, 112].

The prognostic factors for the rate of functional decline of patients with ALS, as assessed with the revised ALS Functional Rating Scale (ALSFRS-R), were recently systematically reviewed [113]. The quality of evidence for the prognostic value of age at onset, site of onset, time from symptom onset to diagnosis, and ALSFRS-R baseline score was graded as “low”, moreover, the prognostic value of initial rate of disease progression, age at diagnosis, forced vital capacity (FVC), presence of FTD, body mass index (BMI), was graded as ‘very low’, mainly due to the limited numbers and the methodological quality of the selected studies. However, most of the prognostic factors for survival previously described also have prognostic value for functional decline, strengthening the relevance of the identified factor [104].

Although it has previously been shown that higher BMI values may slow ALS disease progression, faster decline in ALSFRS-R scores has been reported for patients with a BMI > 30 [114–117]. Since cognitive deficits are considered a negative prognostic factor for ALS, [109, 110] the finding of higher BMI values in ALS-FTD patients may provide a potential explanation for this apparent paradox [118].

Markers related to oxidative stress have also been recently implicated as prognostic factors in ALS: a recent study showed that patients with a lower activity of NADPH oxidase 2 (NOX2), the main reactive oxygen species-producing enzyme, may have a significant increase in survival, [119] while a recent meta-analysis has demonstrated lower levels of uric acid, one of the most important antioxidants in the blood, in ALS patients compared to controls [120].

Potential associations between co-medications and survival in ALS have also been recently explored: the use of proton pump inhibitors has been negatively correlated with survival (HR 1.34, 95 % CI 1.04–1.73), while centrally acting muscle relaxants showed a positive association (HR 0.56, 95 % CI 0.39–0.81) [121], the latter remaining significant after multiple testing correction and potentially explained by an indication bias representing the better prognosis of the upper motor neuron predominant disease variant [122, 123].

## Diagnostic challenges

The diagnosis of ALS is clinical and has been categorized by the El Escorial criteria into various levels of certainty depending on the presence and progressive spread of UMN and LMN signs [124]. Clinical examination, including the careful search for signs of UMN and LMN involvement [125], therefore remains the cornerstone of ALS diagnosis. Moreover, it has recently been suggested that the evaluation of primitive reflexes may also be useful as a simple screening tool for cognitive impairment in ALS [126]. Needle examination, may be extremely useful for the identification of LMN loss and has been incorporated into the revised El Escorial criteria [124]. Although ALS diagnosis is generally fairly simple [127], the false positive rate has been estimated to be as high as eight to 10 %; [128], while the false-negative rate approaches 45 % [129, 130]. Several disorders, often referred to as ALS mimic syndromes, may primarily affect the motor system, simulating ALS. These include Sandhoff disease, adult polyglucosan body disease and Hirayama disease, as highlighted by recent reports [131–133]. In selected cases motor nerve biopsy may be useful for an early differential diagnosis of patients presenting with atypical LMN syndrome [134, 135]. Most importantly, differentiation between MND, in particular progressive muscular atrophy (PMA), and multifocal motor neuropathy (MMN) may be extremely challenging. Although MMN has distinctive neurophysiological features and is often associated with anti-GM1 antibodies [136, 137], a recent large case–control study showed that both PMA and MMN, but not ALS and PLS, are associated with IgM monoclonal gammopathy (8 and 7 %, respectively), suggesting that a subset of patients presenting with PMA share pathogenic mechanisms with MMN [138]. Within this context, nerve ultrasound may provide an additional diagnostic tool, since the ultrasonic finding of focal or multifocal nerve enlargement may strengthen the diagnosis of MMN since is generally not found in ALS [139].

## Advances in neuroimaging

The past few years have seen improvements in anatomical and functional neuroimaging techniques, opening up exciting opportunities to investigate new aspects of the central nervous system structure and function in ALS patients [140]. Efforts are currently directed to phenotypic characterization, identification of brain changes related to clinical outcomes and assessment of their prognostic value.

There are increasing reports of different patterns of grey (GM) and white matter (WM) loss in specific groups of ALS patients [141]. Recent investigations confirm a role for corticospinal tract (CST) metrics in discriminating aggressive forms of ALS from more slowly progressing cases [142–144]. Greater changes along the CST has been observed in bulbar-onset ALS patients compared to limb-onset ones [142]. Patients with faster clinical progression showed more severe cortical thinning of the left precentral gyrus and fractional anisotropy reduction of the CST relative to those with slower rate of progression [143]. A recent study employed Q-ball imaging (QBI), a new diffusion imaging technique which enables a better estimation of intersecting tracts damage than traditional diffusion tensor (DT) MRI, to investigate the extent of WM involvement in ALS [144]. ALS patients showed decreased fiber density and volume, and increased tract length in the corpus callosum and CST relative to healthy controls [144]. In addition, damage to callosal fibers was strongly related to pyramidal impairment and clinical disability. All these findings suggest that an advanced MRI approach has the potential to improve our understanding of neural substrates of clinical impairment in the disease.

Few MRI longitudinal studies have been published on ALS, and the true potential of MRI as a marker for monitoring disease progression has yet to be defined [140]. Longitudinal analyses of small numbers of ALS patients have shown a decrease of cortical thickness in motor, temporal, and fronto-parietal cortices, as well as DT MRI changes in the CST, corpus callosum and frontal regions [141]. A recent study investigating cortical thickness changes over time in different ALS phenotypes found a significant decline of cortical thickness in frontal, temporal, and parietal regions over time [145]. Effects were independent of the clinical subtype, with exception of the precentral gyrus: the LMN ALS variants demonstrated the highest rates of cortical thinning in the precentral gyrus, the UMN-dominant subjects exhibited intermediate rates of atrophy, and the classical ALS patients exhibited no such change. Atrophy of the precentral gyrus in classical ALS indicates a floor effect at the first assessment, resulting in a lack of further atrophy over time [145]. These findings indicate that, while a severe precentral cortical loss is an early sign of classical ALS, atrophy spreads to extra-motor regions over time independently of the phenotypic presentation [145]. Future studies should investigate whether a thinning of the precentral gyrus in LMN ALS variants precedes the transition to classical ALS. A probabilistic fiber tractography study estimated structural connectivity changes after 3 months in patients with ALS [146]. While CST damage did not worsen over time, DT MRI changes were observed in the occipito-temporal tract (linking visual and entorhinal, perirhinal or parahippocampal cortices) [146]. These findings suggest that motor network degeneration starts early (even pre-clinically) in the course of ALS, while extra-motor damage is likely to contribute to the progression of the disease [146].

## Clinical management

Riluzole, an inhibitor of glutamate release has been established as the only and modestly effective disease modifying (neuroprotective) therapy for ALS, extending mean patient survival by 3–6 months [147–149]. Therefore, in the absence of a cure, the cornerstones of the management of patients with ALS are focused on symptom control. These treatments may not only alleviate symptoms but also maintain quality of life and improve survival, with greater benefit for patients managed in specialized, multidisciplinary ALS clinics [150–155]. Symptomatic care in ALS includes a wide range of patient-tailored interventions addressing pain, emotional lability, anxiety and depression, sleep disturbances, constipation and including physiotherapy and adaptive aids. Prevalence of muscle cramps has been estimated to be as high as 44–55 % in ALS patients. As recently reviewed, therapeutic options include quinine sulfate, tetrahydrocannabinol, vitamin B-complex, diltiazem, natridrofuryl oxalate, mexiletine, carbamazepine and leveteracitam as well as non prescription approaches such as muscle stretching [156]. Interestingly, a small case series recently demonstrated that radiotherapy treatment of sialorrhea, which affects up to 25 % of patients [157], may be feasible, efficient and safe, even in patients requiring non-invasive ventilation [158].

Dysphagia and respiratory failure, which is the main cause of mortality for ALS patient, represent two crucial areas of symptomatic interventions for ALS patients.

Given that malnutrition is a negative prognostic factor [159] and aspiration pneumonia one of most feared causes of morbidity and mortality in ALS, current practice guidelines recommend gastrostomy feeding for patients with severe dysphagia [152]. Importantly, a new scale for dysphagia in patients with progressive neuromuscular diseases, including MND, has recently been developed, potentially helping with a more precise evaluation and selection of ALS patients [160]. The two main methods of gastrostomy insertion are percutaneous endoscopic gastrostomy (PEG) and radiologically inserted gastrostomy (RIG), even when per-oral image-guided gastrostomy may also be possible. Even though it has been suggested that RIG may be safer than PEG, in particular in patients with respiratory failure [161, 162], there is to date little evidence to indicate which is the best method and what is the optimum timing for the procedure. Indeed, a recent prospective observational study showed that PEG was a safe procedure even in patients with low forced vital capacity (FVC) [163]. These results were further confirmed by another large prospective cohort study showing that the three methods seemed to be equally safe in relation to survival and procedural complications [164]. However, both studies agree that PEG might be less beneficial when delayed. Further studies, and preferably randomized controlled trials are needed in order to gain better evidences for the nutritional management and gastrostomy in ALS patients.

Respiratory failure is not only the main mortality cause, but may also be the presenting symptom in patients with ALS, in this instance requiring careful differential diagnosis to exclude other neuromuscular and non-neuromuscular disorders [165]. Recommended pulmonary tests are spirometry (including Supine FVC), polysomnography, arterial gas analysis and sniff nasal inspiratory pressure (SNIP), which has recently been shown to be a potential prognostic factor for tracheostomy or death from the early phase of disease [166]. While a recent open-label, randomized controlled trial has shown that addition of diaphragm pacing to standard care was associated with decreased survival in patients with ALS [167], non-invasive ventilation (NIV) has been shown to improve quality of life and survival in patients with ALS [168]. In patients with substantial bulbar impairment efficacy of NIV may be more limited; however, a recent study has demonstrated objective sleep and nocturnal respiratory outcomes in both non-bulbar and bulbar patients [169]. Moreover, NIV has also been reported to significantly increase survival by 19 months in patients with ALS-bulbar onset [170]. In more advanced states, invasive ventilation via tracheostomy is an option for prolongment of survival [171].

## Ethical issues in motor neuron disease

Being a relentlessly fatal disease with no curative therapy currently available, there are many ethical issues in ALS care, from the diagnosis to the latest stages of disease, as recently reviewed [172]. End-of-life discussions, including gastrostomy, NIV and invasive mechanical ventilation are often delayed, potentially leading to unplanned crisis interventions [173]. Although there is no consensus on timing, it is suggested that options for respiratory support and end-of-life issues be discussed if the patient displays symptoms of hypoventilation [174]. Despite the fact that many health-care providers and physicians may find it difficult and stressful [175, 176], ALS patients generally welcome the opportunity to discuss end-of-life issues with their physician [177, 178]. A recent study demonstrated that caregivers and the general public significantly underestimate the QoL of ALS patients and over estimate the patients’ rate of depression. Moreover, the desire to shorten life was significantly lower in ALS patients compared to what healthy subjects thought the patient would wish, emphasizing the need for unbiased patient perspectives in order to secure patient-centred decision making, even if the potential executive and behavioural impairments raises further inevitable questions [179]. A low desire to hasten death and a positive attitude towards life-sustaining treatments were confirmed by another recent study. Of those with an undecided or negative attitude, 10 % changed their attitudes towards life-sustaining treatments during the following year. The same study reported that QoL, depression and social support were not predictors of vital decisions, while the feeling of being a burden was a predictor of decisions against life-supporting treatments [180]. No association between depressive symptoms and euthanasia or physician-assisted suicide (EAS) was reported by a recent prospective study performed in the Netherlands, where EAS has been estimated to be as high as 20 % [181]. Patients’ perspectives concerning PEG and NIV were recently evaluated, showing that the decision-making process is complex, unpredictable and individual, comprising patient-centric factors and external factors, including the roles played by healthcare professionals, family information provided and concepts of timing of interventions [182].

Notably, the recent awareness of potential executive and behavioural impairments in ALS patients represents a further ethical open question, emphasizing the need to develop new methods of neuropsychological assessment suitable to the more advanced stages of disease [179, 183].

## Conclusions

ALS remains a relentlessly progressive and fatal disease and the only approved disease-modifying drug has a modest effect in slowing disease progression. Symptomatic and palliative care, provided in a multidisciplinarycontext, still remains the cornstone of ALS management [148]. However, significant progress has been accomplished in the identification of new genetic factors underlying this disease. Although currently known ALS-related genes still explain a minority of cases, they have helped to unravel the molecular mechanisms of motorneuron degeneration and to identify converging pathways underlying ALS pathogenesis, such as disturbed RNA metabolism or pathological protein aggregation, giving new hope for the development of novel diagnostic and therapeutic approaches.
